# A Bivariate Volterra Series Model for the Design of Power Amplifier Digital Predistorters

**DOI:** 10.3390/s21175897

**Published:** 2021-09-02

**Authors:** Carlos Crespo-Cadenas, María J. Madero-Ayora, Juan A. Becerra

**Affiliations:** Departamento de Teoría de la Señal y Comunicaciones, Escuela Técnica Superior de Ingeniería, Universidad de Sevilla, Camino de los Descubrimientos, s/n, 41092 Seville, Spain; ccrespo@us.es (C.C.-C.); jabecerra@us.es (J.A.B.)

**Keywords:** behavioral modeling, digital predistortion, nonlinear model identification, power amplifier linearization, Volterra series

## Abstract

The operation of the power amplifier (PA) in wireless transmitters presents a trade-off between linearity and power efficiency, being more efficient when the device exhibits the highest nonlinearity. Its modeling and linearization performance depend on the quality of the underlying Volterra models that are characterized by the presence of relevant terms amongst the enormous amount of regressors that these models generate. The presence of PA mechanisms that generate an internal state variable motivates the adoption of a bivariate Volterra series perspective with the aim of enhancing modeling capabilities through the inclussion of beneficial terms. In this paper, the conventional Volterra-based models are enhanced by the addition of terms, including cross products of the input signal and the new internal variable. The bivariate versions of the general full Volterra (FV) model and one of its pruned versions, referred to as the circuit-knowledge based Volterra (CKV) model, are derived by considering the signal envelope as the internal variable and applying the proposed methodology to the univariate models. A comparative assessment of the bivariate models versus their conventional counterparts is experimentally performed for the modeling of two PAs driven by a 30 MHz 5G New Radio signal: a class AB PA and a class J PA. The results for the digital predistortion of the class AB PA under a direct learning architecture reveal the benefits in linearization performance produced by the bivariate CKV model structure compared to that of the univariate CKV model.

## 1. Introduction

Efficiency is of paramount importance in modern wireless communication systems, affecting different aspects of their implementation. On the one hand, high spectrally efficient modulation schemes are required, such as Orthogonal Frequency Division Multiplexing (OFDM), in order to satisfy the ever-increasing demand for data rate and connectivity. On the other hand, the evolution towards more sustainable communications involves the search for energy-efficient transceivers, with the power amplifier (PA) being the most critical subsystem of the transmitter in terms of power consumption. There is an inherent trade-off between linearity and power efficiency in PAs [1]. Applying a certain backoff from the PA saturation point in order to limit the nonlinear distortion is a simple option with the cost of reducing its efficiency. In contrast to this, the use of linearization techniques such as digital predistortion (DPD) can help to tip the balance in favor of efficiency [2]. Nevertheless, the design of a DPD relies on a behavioral model that veraciously resembles the inverse function of the PA.

Behavioral models of PAs have evolved from the traditional characterization based on the AM/AM and AM/PM static curves to the modern discrete-time behavioral models. The latter are tailored to take into account not only nonlinear distortion but also memory effects. The relevance of memory effects is emphasized by the waveforms based on OFDM, with scalable numerology and augmented bandwidths, which are defined by the fifth generation New Radio (5G-NR) air interface. As the discrete-time models are better suited for the implementation of DPDs, the correct selection of the model structure plays a key role to optimize the linearization performance.

Diverse approaches can be adopted for the discrete-time modeling of nonlinear systems, such as multidimensional polynomial filters or artificial neural networks. Volterra series [3,4] is also a useful mathematical tool for the analysis of nonlinear microwave and wireless systems [5]. It can be considered the extension of Taylor series to nonlinear systems with memory, whose *n*-order terms are obtained by means of a nonlinear generalization of the convolution integral that is commonly employed for the study of linear time invariant (LTI) systems. Volterra series produce behavioral models that are linear with respect to the parameters, thus allowing the use of linear regression methods for their identification [6]. With the purpose of modeling wireless nonlinear systems that are driven by communications signals, Volterra series expansions are considered in a discrete, time-sample form. The input complex envelope is sampled at time t=kT, with *T* being the sampling period and k∈Z, thus producing the discrete-time sequence x(k).

Based on the baseband equivalent Volterra model [7], the discrete-time amplifier output at the fundamental frequency zone is described by a linear combination of regressors. These regressors are given by odd-order monomials of the discrete-time input complex envelope x(k) and its conjugate $x^{*}\left(k\right)$ with different delays that are denoted by $q,q_{1},q_{2},$ etc. If this univariate Volterra series is truncated to a maximum nonlinear order and memory depth, the resulting structure is denoted as the full Volterra (FV) model. Pioneering publications of baseband DPDs have also proposed the memory polynomial MP [8] and the generalized memory polynomial GMP models [9]. The MP and GMP models involve terms with the cross product of the signal and its exponentiated envelope, e.g., the MP model contains terms

(1) $${x\left(k-q\right)|x\left(k-q\right)|}^{n-1}$$

and the GMP model contains terms

(2) $$x\left(k-q_{1}\right){\left|x\left(k-q_{1}-q_{2}\right)\right|}^{n-1}$$

for all *n*, odd and even. Since only terms with odd *n* values are expected in a conventional FV model, some researchers have been reticent with regard to the use of *n*-even terms notwithstanding their beneficial effect on the model fitting capabilities [10,11]. Apart from these widely popular heuristic approaches, several models derived from previous knowledge of the device have been proposed [12,13,14]. In particular, the kernel structure of the model published in [14] was derived on basis of the available equivalent-circuit information of a typical FET device with thermal power feedback. This circuit-knowledge based Volterra model is referred to here to as the CKV model. Like the FV model, the CKV model contains only conventional odd-order terms, which impose a limitation to its accuracy and a pertinent question arises: can the univariate FV and CKV models also be made richer with some kind of “beneficial” circuit-knowledge derived terms?

In the present paper, a novel structure with beneficial terms is demonstrated following a procedure based on a circuit knowledge perspective. First, an internal mechanism which is responsible for the signal envelope generation is identified and this new subnetwork is included in the CKV approach to augment its kernel structure. A basic example of the mentioned enhancement was advanced in [15], for a simple case of the bivariate CKV model due to the space limitations in a conference paper. In addition to the benefits of these terms in a general CKV model, this new communication also reveals the enhancement of the FV model when the bivariate structure is included.

The existence of the internal envelope waveform provides a rationale to its use as a second control signal in a double Volterra series proposal [16]. In Section 2, a general bivariate model is derived from the conventional baseband FV model, the bivariate FV, with an enhanced regressor set. Additionally, the demonstration goes further into the PA circuit knowledge to provide a less complex bivariate Volterra series model, the bivariate CKV. Compressed-sensing techniques are applied to identify the optimum set of active regressors of the proposed models in different experimental scenarios. In Section 3, the modeling performance of this approach is contrasted with the conventional Volterra models in the case of two amplifiers, a class AB and a class J. For comparison, the results for the GMP and the simplified model presented in [15] are also included. Finally, the aptitude of the bivariate models in the design of DPDs to linearize PAs is considered in Section 4 and a summary is presented in the last section.

## 2. A Bivariate Volterra Series Perspective

Conventional behavioral models express the PA output depending on the input RF signal

(3) $$\tilde{x}\left(t\right)=ℜ\left\{x\left(t\right)e^{j\omega_{c}t}\right\},$$

i.e., the PA is articulated as a univariate nonlinear system. This section focus on behavioral models described under a Volterra series perspective. Particularly, the general FV model and the model based on circuit knowledge CKV, and the methodology to derive the respective enhanced models are covered.

### 2.1. Conventional PA Models

#### 2.1.1. The Full Volterra Model

The FV model is the discrete-time and truncated version of the baseband model [7]. Compared to other structures of the Volterra series group, this model incorporates the more ample regressors set and its output is represented by a sum of nonlinear terms with n=2p−1, i.e., only odd-order terms. The input/output relationship is written as

(4) $$y\left(k\right)=\sum_{p=1}^{P}\sum_{q_{2p-1}=0}^{Q_{2p-1}}h_{2p-1}\left(q_{2p-1}\right)\prod_{r=1}^{p}x\left(k-q_{r}\right)\prod_{r=p+1}^{2p-1}x^{*}\left(k-q_{r}\right),$$

where $h_{2p-1}\left(q_{2p-1}\right)$ is the Volterra kernel with order n=2p−1, $q_{n}={\left[q_{1},q_{2},\ldots ,q_{n}\right]}^{T}$ is a vector of delays of the *n*-th order term, $Q_{n}={\left[Q_{n},Q_{n},\ldots ,Q_{n}\right]}^{T}$ is the vector of maximum delays, and

(5) $$\prod_{r=1}^{p}x\left(k-q_{r}\right)=x\left(k-q_{1}\right)x\left(k-q_{2}\right)\cdots x\left(k-q_{p}\right).$$

#### 2.1.2. The Circuit-Knowledge Based Volterra Model

**Figure 1:**
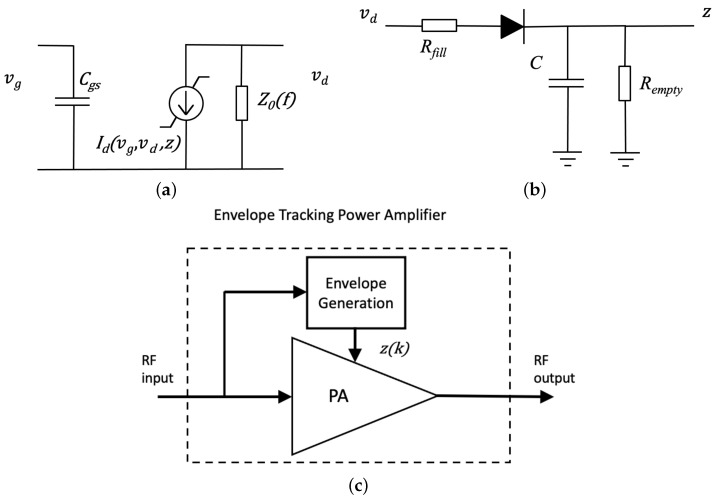
Simplified layouts of a single-FET amplifier (**a**), the associated charge trapping subcircuit (**b**) and the envelope tracking PA (**c**). The following elements are included: gate voltage $v_{g}$, drain voltage $v_{d}$, internal variable *z*, drain current $I_{d}$, gate-to-source capacitance $C_{gs}$, frequency-dependent load impedance $Z_{0}\left(f\right)$, capacitance *C*, and resistances $R_{fill}$ and $R_{empty}$.

The CKV model, whose detailed methodology is published in [14], is a proposal to identify the kernel structure under the premise of the PA circuit-model knowledge. The reasoning here can be reduced to essentials with an analysis based on the simple PA schematic of Figure 1a, as well as the addition of the electrothermal subnetwork (not shown in the figure). The approach contemplates the derivation of the nonlinear transfer functions including the gate voltage-drain voltage $v_{g}$-$v_{d}$ cross-terms of the nonlinear drain current source and the junction temperature rise acting as the internal variable *z*. The deduction complies with a standard Volterra series approach to demonstrate the baseband CKV model, given by

(6) $$y\left(k\right)=\sum_{p=1}^{P}\sum_{q_{p}=0}^{Q_{p}}h_{2p-1}\left(q_{p}\right)x\left(k-q_{1}\right)\prod_{r=2}^{p}{\left|x\left(k-q_{1}-q_{r}\right)\right|}^{2},$$

where $h_{2p-1}\left(q_{p}\right)$ is the Volterra kernel with (odd) order n=2p−1, $q_{p}={\left[q_{1},q_{2},\ldots ,q_{p}\right]}^{T}$ is a vector of delays of the *n*-th order term, and $Q_{p}={\left[Q_{p},Q_{p},\ldots ,Q_{p}\right]}^{T}$ is the vector of maximum delays. By definition,

(7) $$\prod_{r=2}^{p}{\left|x\left(k-q_{1}-q_{r}\right)\right|}^{2}=1,$$

for p=1. Notice that the CKV model in (6) contains a subset of the FV regressors.

### 2.2. Bivariate Volterra Models

PA models implicitly contemplate a nonlinearity dependent on a single variable, the input RF signal. Actually, the FET current source depends on several variables, for example, the gate and drain voltages, $v_{g}$ and $v_{d}$. More complete transistor models put forward the presence of an additional mechanism generating an internal variable, such as the aforementioned electrothermal subcircuit.

In the search of new, possibly beneficial, regressors let us recall the charge-trapping internal mechanism, which is the cause of drain-lag effects in FET devices [17]. From observing the simplified diagram of a one-trap model shown in Figure 1b, it is evident that this subcircuit basically works as an envelope detector. Therefore, the charge-trapping subcircuit generates a new internal variable (the real-valued signal envelope) that additionally controls the current source of the transistor. Another example of an amplifier with a mechanism generating a secondary signal is the high-efficiency envelope tracking (ET) PA [18]. In the ET-PA configuration, shown in Figure 1c, the RF envelope is detected and amplified to provide a dynamically adjusted supply voltage to the basic PA.

**Figure 2:**
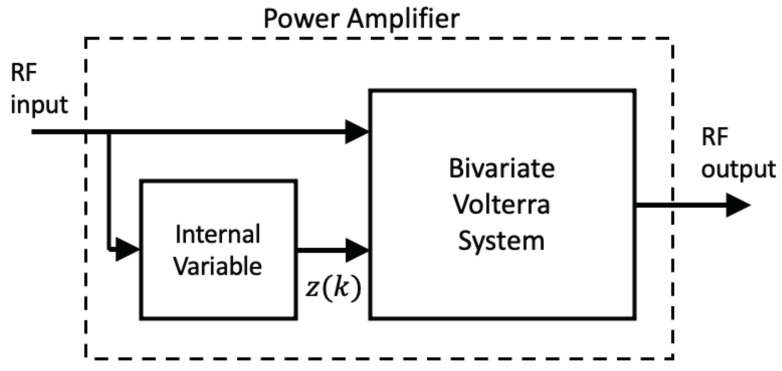
A bivariate Volterra approach to PAs with an internal variable generation.

The two examples discussed above fit the schematic representation of Figure 2, where the internally generated envelope acts as the second input to a bivariate block. If this block is a bivariate Volterra system and the two inputs are the RF signal $\tilde{x}\left(k\right)$ and the envelope z(k), the discrete-time output can be expressed as a double Volterra series [16].

The output of the bivariate Volterra system is organized into three groups: the first group is a univariate Volterra series dependent on the RF signal $\tilde{x}\left(k\right)$, the second group is another univariate Volterra series dependent on the envelope z(k), and the third group contains cross products of $\tilde{x}\left(k\right)$ by z(k). Therefore, the RF output is given by

(8) $$\begin{array}{c} \tilde{y}\left(k\right)={\hat{H}}_{a,0}\left[\tilde{x}\left(k\right)\right]+{\hat{H}}_{b,0}\left[z\left(k\right)\right]+\\ \;\;\;\;\;\;\;\;\;+\sum_{n=1}^{N}\sum_{m=1}^{M}\sum_{q_{n}=0}^{Q_{n}}\sum_{q_{m}^{\prime }=0}^{Q_{m}^{\prime }}h_{n,m}\left[q_{n},q_{m}^{\prime }\right]\prod_{r=1}^{n}\tilde{x}\left(k-q_{r}\right)\prod_{s=1}^{m}z\left(k-q_{s}^{\prime }\right), \end{array}$$

where ${\hat{H}}_{a,0}\left[\cdot \right]$ and ${\hat{H}}_{b,0}\left[\cdot \right]$ are univariate Volterra operators. The third group of sums contains the bivariate Volterra kernels $h_{n,m}\left[q_{n},q_{m}^{\prime }\right]$ and cross products of the RF input signal $\tilde{x}\left(k-q_{r}\right)$ by the envelope $z(k-q_{s}^{\prime })$. The vector of envelope delays and the vector of maximum delays are denoted as $q_{m}^{\prime }$ and $Q_{m}^{\prime }$, respectively. Univariate Volterra models are also referred to here as conventional Volterra models.

#### 2.2.1. The Bivariate FV Model

Since we are interested in the fundamental frequency zone, the baseband equivalent of the first group in (8) reduces to a model dependent on the complex envelope x(k). Recalling that the envelope z(k) is a baseband real-valued variable, the second group of terms do not contribute to the fundamental frequency zone and can be neglected. Summing up, the output complex envelope can be written as

(9) $$y\left(k\right)=H_{a,0}\left[x\left(k\right)\right]+\sum_{m=1}^{M}\sum_{q_{m}^{\prime }=0}^{Q_{m}^{\prime }}H_{a,m}\left[x\left(k\right)\right]\prod_{s=1}^{m}z\left(k-q_{s}^{\prime }\right),$$

where $H_{a,0}\left[x\left(k\right)\right]$ is the conventional FV model (4) and the second group of terms is the result of multiplying the baseband Volterra model by monomials of the form $z\left(k-q_{1}^{\prime }\right)\cdots z\left(k-q_{m}^{\prime }\right)$. This perspective discloses nonexplored modeling alternatives based on the specific conventional Volterra model used for $H_{a,0}\left[x\left(k\right)\right]$, covering from the FV model, with a richer regressors set, to simpler models, with pruned sets of regressors. The relationship (9) is referred to as the bivariate FV (bi-FV) model.

#### 2.2.2. The Bivariate CKV Model

Assuming the same line of reasoning based on circuit knowledge of the CKV model (6) to illustrate the present approach and recalling that z(k)=|x(k)|, the relationship (9) yields

(10) $$\begin{array}{c} y\left(k\right)=\sum_{p=1}^{P}\sum_{q_{p}=0}^{Q_{p}}h_{2p-1}\left(q_{p}\right)x\left(k-q_{1}\right)\prod_{r=2}^{p}{\left|x\left(k-q_{1}-q_{r}\right)\right|}^{2}+\\ \;\;+\sum_{m=1}^{M}\sum_{q_{m}^{\prime }=0}^{Q_{m}^{\prime }}\sum_{p=1}^{P}\sum_{q_{p}=0}^{Q_{p}}h_{2p-1}\left(q_{p}\right)x\left(k-q_{1}\right)\prod_{r=2}^{p}|x\left(k-q_{1}-q_{r}\right){|}^{2}\prod_{s=1}^{m}\left|x\left(k-q_{s}^{\prime }\right)\right|. \end{array}$$

Henceforth, the representation (10) is referred to as the bivariate CKV (bi-CKV) model. Contrary to the second sum, the first sum of this expression is made up of conventional odd-order Volterra terms.

Observe that, in the particular case that the indices satisfy $q_{1}=l$, $q_{r}=m$ for all r≥2, and $q_{s}^{\prime }=l+m$, the second sum incorporates the beneficial GMP terms ${x\left(k-l\right)|x\left(k-l-m\right)|}^{2p+1}$.

### 2.3. Model Order Reduction

Not all the regressors of the bivariate model are significant and a first step to extract the coefficients is the identification of the active regressors set. Starting with the whole stock of regressors, the procedure follows a doubly orthogonal matching pursuit (DOMP) algorithm to search the most significant regressors [19,20]. The active regressors set is delimited following a Bayesian information criterion (BIC) to provide a reduced-order model. The BIC rule stops the search by adding to the normalized mean squared error (NMSE) a penalty term to avoid overfitting [19]

(11) $$\mathrm{BIC}\left(n_{a}\right)=\mathrm{NMSE}\left(n_{a}\right)+\frac{n_{a}}{N_{s}}10\log\left(2N_{s}\right),$$

where $n_{a}$ is the number of active regressors and $N_{s}$ is the number of signal samples used in the estimation. In that form, the optimum set of active regressors is identified, and the result is a pruned model. If the bivariate regressors are excluded in the initial stock, the search identifies a univariate Volterra model with limited precision. Then, it is possible to compare the capabilities of the two optimized models, the conventional and the bivariate Volterra models.

The superior performance of models with *n*-even terms, i.e., bivariate Volterra regressors, has been already advanced for MP models [10,11]. It is interesting to note here that the ET-PA fits the schematic of Figure 2 due to the presence of a mechanism to generate the envelope for the modulated supply voltage. A significant accuracy improvement has been reported in an experimental work when the model incorporates the GMP bivariate Volterra terms [21]. In this work, we also demonstrate the performance improvement attained with the bivariate Volterra model for two cases, a class AB and a class J PA.

## 3. PA Modeling Performance

**Figure 3:**
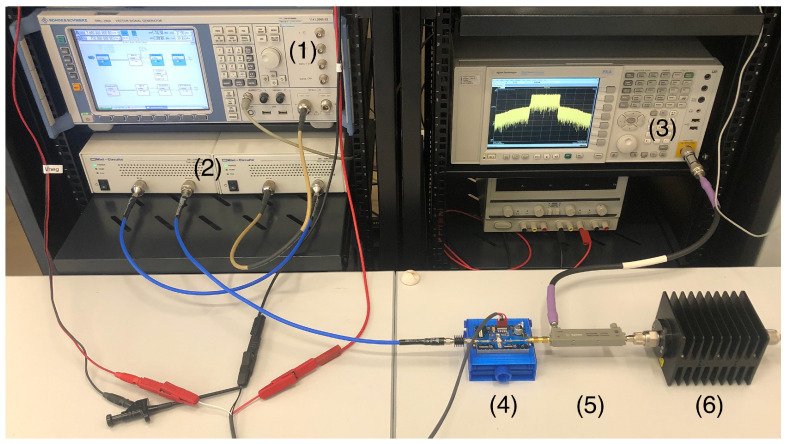
Photograph of the experimental setup composed of (1) a vector signal generator, (2) two cascaded preamplifiers, (3) a vector signal analyzer, (4) the class AB PA under test, (5) a directional coupler, and (6) an attenuator terminated with a 50Ω load.

The experimental setup shown in Figure 3 was employed for the comparative assessment of the proposed approach with bivariate models versus the use of their conventional counterparts. The probing signal, designed according to the 5G-NR standard [22] with a 30 MHz bandwidth, was created by an SMU200A vector signal generator (VSG) from Rohde & Schwarz with built-in arbitrary waveform generator. With a peak-to-average power ratio (PAPR) of 10.5 dB, it contained over 180,000 samples, corresponding to a sampling frequency of 92.16 MSample/s. The VSG was followed by two cascaded Mini-Circuits TVA-4W-422A+ preamplifiers in order to drive the PA under test into a mildly nonlinear operation, while keeping the behavior of the modulator sufficiently linear.

Two different PAs were employed in the experiments. The first PA under test was the evaluation board of a class AB amplifier based on the CGH40010 GaN HEMT from Cree Inc. (Durham, NC, USA), operated with a drain-to-source current of about 200 mA at 3.6 GHz and an average output power of 27.4 dBm. The second PA under test was a continuous-mode class J amplifier designed over the CGH35015F GaN HEMT from Cree Inc., providing an average output power of 26 dBm at 850 MHz. The output of the PAs was fed to a PXA-N9030A vector signal analyzer (VSA) from Keysight Technologies through a directional coupler and an attenuator in order to avoid introducing undesired distortion from the equipment. The RF output signal was downconverted to baseband and acquired in the VSA, where the measurement dynamic range was optimized by averaging 300 repetitions of the measured signal. Finally, the signals were time-aligned in order to synchronize the input and output data sets.

First, we compare the different modeling performances of the CKV, FV, bi-CKV and bi-FV models for the class AB PA. An important drawback of the bi-FV model compared to the bi-CKV model is its greater complexity in terms of number of regressors (and coefficients, correspondingly). In order not to go beyond the limits of computing capacity when predicting the PA output, all the models were selected with a ninth nonlinear order and a memory of three samples. Furthermore, the memory is considered only for regressors with nonlinear order below 7 in the case of the FV (univariate and bivariate) models.

**Figure 4:**
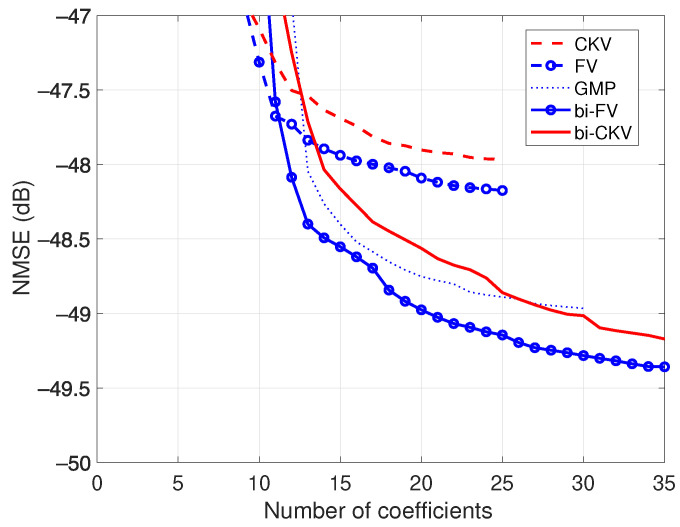
NMSE results of the conventional and the bivariate Volterra models, for a class AB power amplifier operated at 3.6 GHz with an average output power of 27.4 dBm.

The reduction in the NMSE between the modeled and the measured output of the class AB PA as the DOMP algorithm incorporates new regressors to the active set is shown in Figure 4. For the conventional CKV model (plotted with a dashed line), the best NMSE according to the BIC rule is about −48 dB. An FV model with the same order and memory improves the NMSE (dashed line with circles), an expected result considering its richer regressors set. On the other hand, the improvement is marginal, indicating that the CKV model is quite appropriate despite its sparseness. For comparison purposes, the GMP model curve is also plotted (dotted line), showing the effect of its beneficial terms. The better accuracy of the bi-CKV model (10) (displayed in the figure with a solid line) and the further NMSE improvement obtained with the bi-FV model (solid line with circles) are clear results of the bivariate terms.

The NMSEs of the conventional CKV and FV models do not reach the level of error of the corresponding bivariate models, even in the case that more regressors are incorporated, evidencing that without bivariate terms, the stock of regressors is insufficient.

Since the extension of nonlinear order and memory length is impracticable for the bivariate FV model, in the following the focus will be put in the comparison of the conventional and bivariate CKV models with optimized order and memory.

### 3.1. Signal Alignment

**Figure 5:**
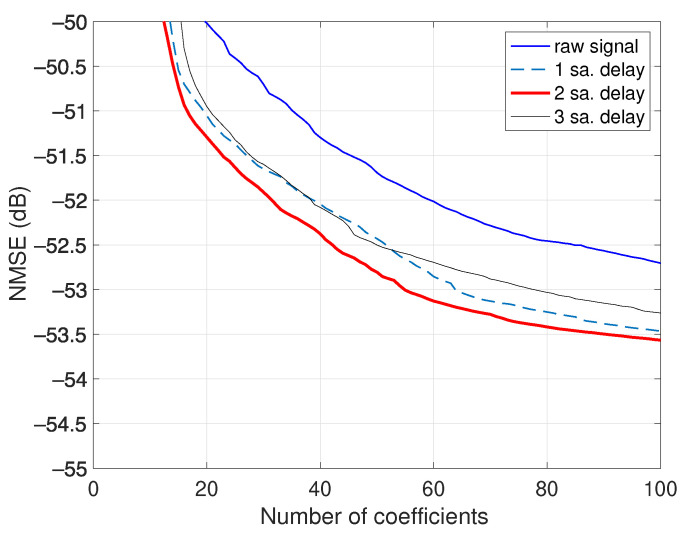
NMSE results of the bi-CKV models for different input–output alignments for a class AB power amplifier operated at 3.6 GHz with an average output power of 27.4 dBm. The NMSE is optimized with a delay of 2.

Notice that a perfect time alignment of the envelope z(k) and the input x(k) is assumed in the bi-CKV model (10), i.e., the envelope delays $q_{m}^{\prime }\geq 0$. In case that the envelope is advanced with respect to x(k), the lower limit of $q_{m}^{\prime }$ must be a negative index. A second presumption is a perfect synchronization of the acquired output y(k) and the input. This signal alignment is commonly performed with a postprocessing procedure based on the best cross correlation between y(k) and x(k) [6]. An error in the timing coincidence can yield a raw signal $\hat{y}\left(k\right)$ advanced in time, and the bi-CKV model would require noncausal terms with a dependence on “future” input samples. Alternatively, it is possible to refine the synchronization by delaying the coarse aligned signal $\hat{y}\left(k\right)$, the number of samples necessary to optimize the NMSE. The procedure is illustrated in Figure 5, where the NMSE of a bi-CKV model with ninth-order and 10 samples of memory length are plotted for the raw output signal of the class AB PA under the same operating conditions. When repeating the modeling with the signal delayed one sampling time, the NMSE improvement is appreciable. The optimum result is given for a delay of two samples, with an error reduction of about 1 dB.

### 3.2. Comparison to a Simplified bi-CKV

In the conference paper [15], a simplified case of the bi-CKV model (10) was proposed assuming $q_{2}=q_{3}=q_{4}=\cdots$ and $q_{1}^{\prime }=q_{2}^{\prime }=q_{3}^{\prime }=\cdots =q_{1}+q_{2}$. When grouping separately the terms with the envelope raised to an even power and the terms with the envelope raised to an odd power, the output is given by

(12) $$\begin{array}{c} y\left(k\right)=\sum_{n=1}^{N}{}^{\prime }\sum_{q_{2}=0}^{Q_{2}}h_{n}\left(q_{1},q_{2}\right)x\left(k-q_{1}\right){\left|x\left(k-q_{1}-q_{2}\right)\right|}^{n-1}+\\ \;\;\;\;\;\;\;\;\;+\sum_{n=2}^{N}{}^{\prime \prime }\sum_{q_{2}=0}^{Q_{2}}h_{n}\left(q_{1},q_{2}\right)x\left(k-q_{1}\right){\left|x\left(k-q_{1}-q_{2}\right)\right|}^{n-1}. \end{array}$$

**Figure 6:**
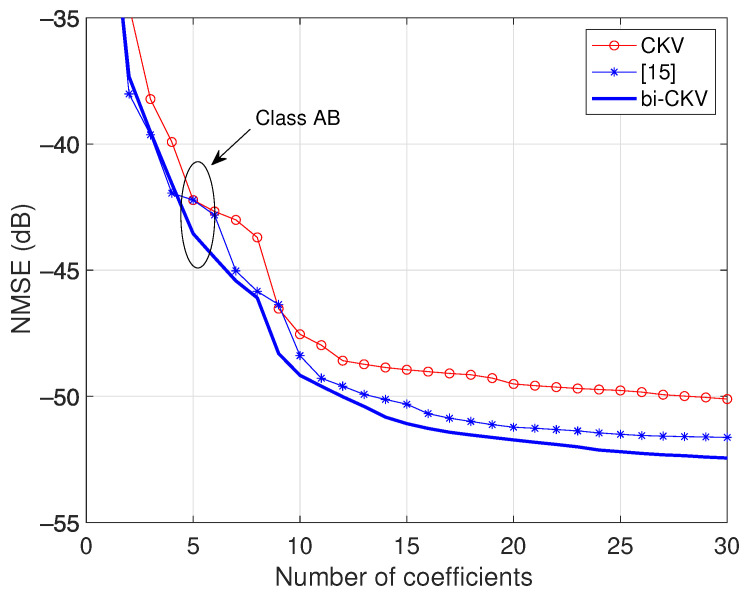
Comparison of NMSE for the bi-CKV model and the results published in [15] applied to a class AB power amplifier operated at 3.6 GHz with an average output power of 27.4 dBm.

The prima (${\;}^{\prime }\;$) indicates that only odd *n* indices are included in the first sum and the double prima (${\;}^{\prime \prime }\;$) indicates that only even *n* indices are included in the second sum. Under this perspective, the GMP model can be considered a particular case of the bivariate CKV model. In Figure 6, the results of the model published in [15] are plotted with asterisks marks, exhibiting a superior performance compared to the univariate CKV model. The NMSE of the present bi-CKV model, indicated with a solid line in the same figure, displays an appreciable further improvement.

**Figure 7:**
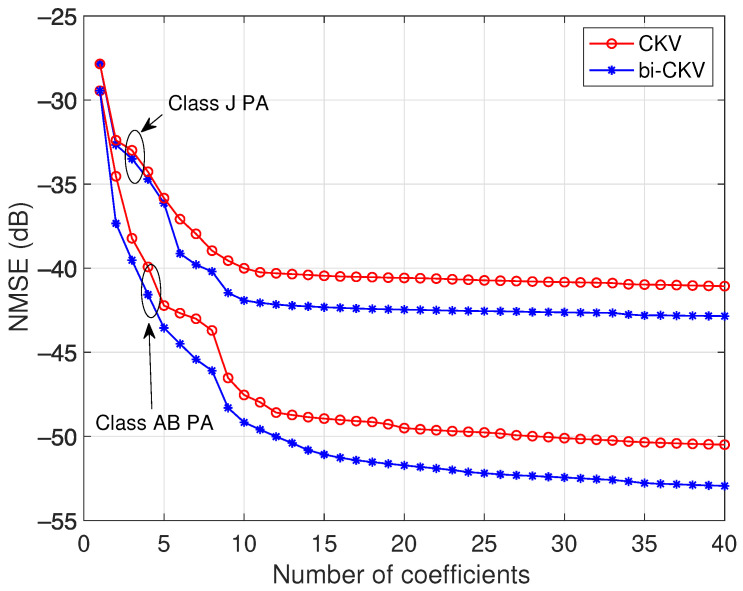
NMSE results of the CKV and the bi-CKV models, for a class AB PA operated at 3.6 GHz with an average output power of 27.4 dBm and a class J PA operated at 850 MHz with an average output power of 26 dBm.

The NMSE results for a class AB and a class J amplifiers are depicted in Figure 7. The evolution of the error versus the number of coefficients that the DOMP algorithm adds to the active regressors set under the restriction of the BIC rule demonstrates an optimum number between 15 and 20 regressors for the class AB PA model. The behavior of the class J PA is more complex—it exhibits not only gain compression but also a notable gain expansion for low power levels, and the corresponding optimum model contains a number between 20 and 25 regressors fixed by the BIC. The data show that conventional models are outperformed by the bivariate models in the two PA cases. It can be observed that even in the case that we continue adding more coefficients, the conventional models do not improve the NMSE. This fact indicates that, without bivariate terms, their stock of regressors lacks the necessary terms to reach a better precision.

## 4. Application of the Bivariate Model to DPD

With the aim of highlighting the enhancement in linearization capabilities after the inclusion of bivariate model terms, a direct learning architecture (DLA) [23] was executed in the DPD of a commercial PA. The experimental setup of Figure 3 was employed for that purpose, where the selected operation point for the class AB PA was characterized by an average output power of 31.2 dBm (39.8 dBm of peak output power) and a gain compression of 2.8 dB.

**Figure 8:**
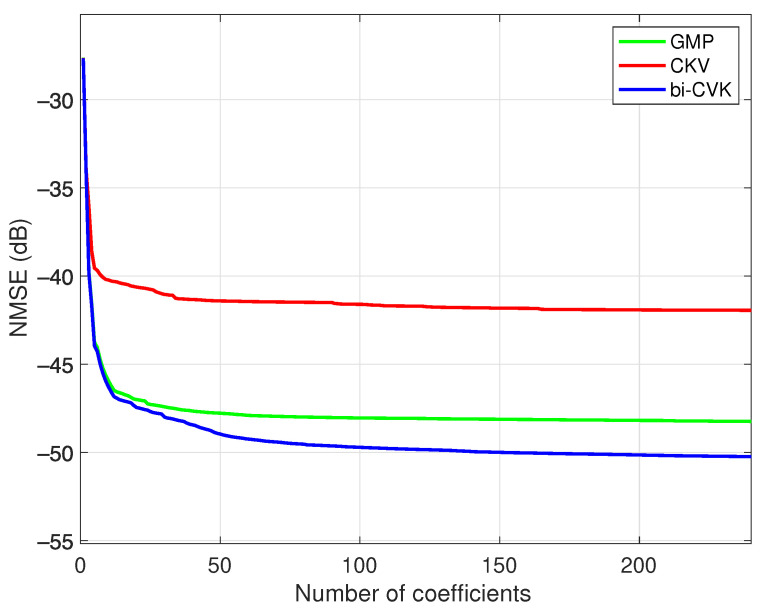
Identification NMSE in the search of the optimum DPD structure with respect to the number of selected components for the GMP and the CKV models in its conventional and bivariate versions. A class AB PA at 3.6 GHz was employed with an operation point characterized by an average output power of 31.2 dBm.

First, a search of the optimum structure with the DOMP technique was executed over the GMP and the CKV models in the conventional and bivariate forms. The training signals for this experiment were the input and output waveforms of the PA without DPD. In order to have a comparable set of initial regressors, the models were set to seventh order and maximum memory depth of seven taps. The initial numbers of coefficients for the GMP, the CKV and the bi-CKV models were 248, 328 and 812, respectively. The learning curve of the DOMP algorithm with respect to the number of coefficients is shown in Figure 8, where the expectable decreasing error with the number of coefficients shows the best modeling capabilities in the bivariate CKV structure. The enhancement with respect to the conventional version motivates the increase in number of initial coefficients. The bi-CKV model also shows an enhancement with respect to the GMP model, since it represents a richer regressor structure. The BIC was executed over the sweep in number of components to set the optimum number of coefficients of 99, 165 and 210 for the GMP for the CKV and the bi-CKV models, respectively. These selected regressors were fixed and a DPD was designed based on them in a DLA scheme.

**Figure 9:**
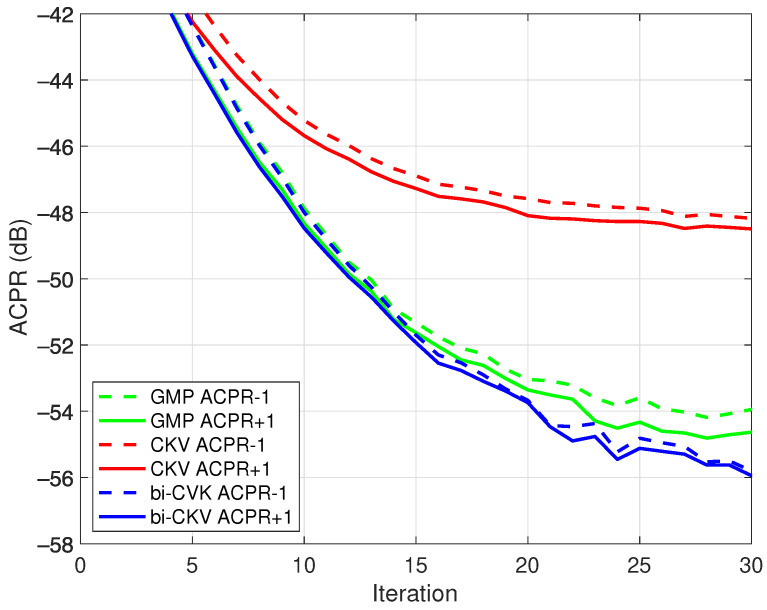
Evolution of the ACPR in the first two adjacent channels with the iterations in a direct learning scheme for class AB PA operated at 3.6 GHz with an average output power of 31.2 dBm.

The DPD training was executed for 30 iterations with a step size of μ=1/2. In this experiment, the linearization performance was measured by means of the adjacent channel power ratio (ACPR) of the output signal. The linearization NMSE exhibits a similar behavior and it is omitted for simplicity. The DPD performance with respect to DLA iterations is shown in Figure 9, where the linearization capabilities of each algorithm in comparison follow a similar trend closely related to the search of the optimum structure.

**Figure 10:**
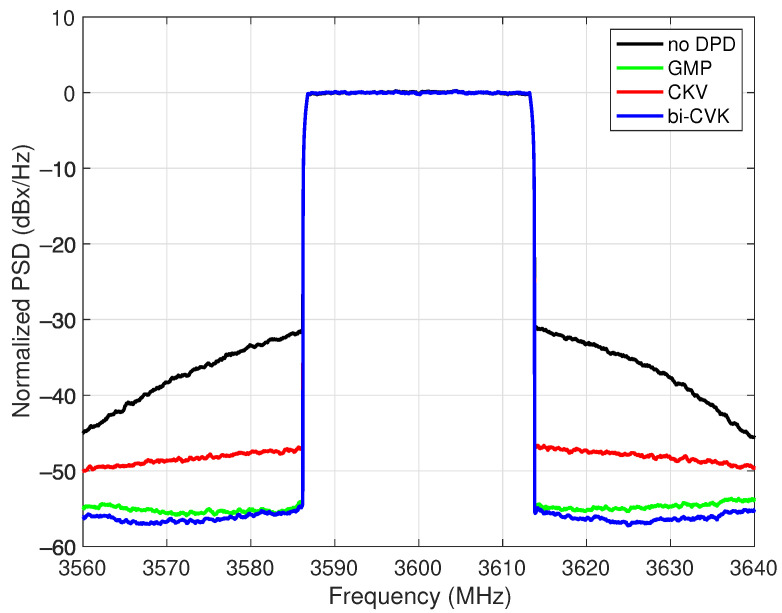
Normalized power spectral densities (PSDs) of the output signal without DPD and with the models in comparison for class AB PA operated at 3.6 GHz with an average output power of 31.2 dBm.

Finally, once the DPD coefficients were trained and fixed, a new validation signal was set as the input to feature the algorithm’s linearization performance by their ability to work with signals not used in the model identification stage. The normalized power spectral densities (PSDs) of the output waveform without DPD and with the benchmarked algorithms are presented in Figure 10. This figure reveals the effectiveness of the DPD exhibiting a spectral regrowth reduction in accordance to the attained performance in finding the model structure and training the DPD coefficients.

## 5. Conclusions

A bivariate proposal for PA modeling has been presented in this work. The approach considers an internally generated variable which operates as the second input to a bivariate Volterra system, giving a PA output expressed as a double Volterra series. In the present exposition, the beneficial terms with the envelope raised to an odd power respond to a bivariate Volterra model structure and are formally obtained following a procedure based on previous circuit model knowledge. Starting from a general representation, the particular case of the GMP model seen as a bivariate Volterra model is demonstrated.

Experimental data show that the modeling capabilities of a reduced-order bivariate Volterra model applied to both a class AB and a class J PAs outperform those of the univariate Volterra model. Finally, the linearization results for a 30 MHz 5G-NR signal and the class AB PA operating with an average output power of 31.2 dBm (gain compression of 2.8 dB) indicate that the NMSE and ACPR for the upper and lower adjacent channels achieved by the conventional CKV model are −41.9 dBc and −48.5/−48.1 dBc, respectively, compared to the values of −47.0 dB and −54.8/−54.2 dBc for the GMP model, and values of −47.4 dB and −55.7/−55.5 dBc for the bivariate CKV model.

Further development of the present approach, constructed with more complex models (e.g., the FV model), can provide bivariate models with a richer set of regressors and a reasonably improved performance.

## Data Availability

The data presented in this study are available on reasonable request from the corresponding author.
